# Sirt1 Activation by Post-ischemic Treatment With Lumbrokinase Protects Against Myocardial Ischemia-Reperfusion Injury

**DOI:** 10.3389/fphar.2018.00636

**Published:** 2018-06-15

**Authors:** Yi-Hsin Wang, Shun-An Li, Chao-Hsin Huang, Hsing-Hui Su, Yi-Hung Chen, Jinghua T. Chang, Shiang-Suo Huang

**Affiliations:** ^1^Institute of Medicine, Chung Shan Medical University, Taichung, Taiwan; ^2^Superintendent Office, Yuanli Lee’s General Hospital, Lee’s Medical Corporation, Miaoli, Taiwan; ^3^Department of Internal Medicine, Dajia Lee’s General Hospital, Lee’s Medical Corporation, Taichung, Taiwan; ^4^Department and Institute of Pharmacology, School of Medicine, National Yang-Ming University, Taipei, Taiwan; ^5^Graduate Institute of Acupuncture Science and Research Center for Chinese Medicine and Acupuncture, China Medical University, Taichung, Taiwan; ^6^Department of Photonics and Communication Engineering, Asia University, Taichung, Taiwan; ^7^Department of Pharmacology and Institute of Medicine, Chung Shan Medical University, Taichung, Taiwan; ^8^Department of Pharmacy, Chung Shan Medical University Hospital, Taichung, Taiwan

**Keywords:** lumbrokinase, cardioprotection, ischemia, reperfusion, Sirt1

## Abstract

Lumbrokinase is used as an oral supplement to support and maintain healthy cardiovascular function, and to treat cardiovascular diseases in clinical for more than 10 years. Up until now, the mechanism of the cardioprotective effects of post-ischemic treatment with lumbrokinase has remained unclear. We therefore investigated the signaling pathways involved in the amelioration of myocardial ischemia-reperfusion (I-R) injury in rats treated with lumbrokinase 20 min after myocardial ischemia. Compared to vehicle-treated rats, post-ischemic treatment with lumbrokinase was associated with significant reductions in myocardial I-R-induced arrhythmias and myocardial damage, and an improvement in cardiac function. Moreover, lumbrokinase significantly upregulated levels of silent information regulator 1 (Sirt1). In addition, lumbrokinase significantly increased manganese-dependent superoxide dismutase expression, decreased Cleaved-Caspase-3 expression, and induced deacetylation of FoxO1. On the other hand, lumbrokinase also significantly downregulated levels of succinate dehydrogenase, cytochrome c oxidase, nuclear factor kappa B (NF-κB) and elevated levels of microtubule-associated protein light chain 3. Notably, the cardioprotective effects of lumbrokinase were abolished by administration of the specific Sirt1 inhibitor EX527. These findings demonstrate that post-ischemic treatment with lumbrokinase attenuates myocardial I-R injury through the activation of Sirt1 signaling, and thus enhances autophagic flux and reduces I-R-induced oxidative damage, inflammation and apoptosis.

## Introduction

Ischemic heart disease (IHD) is a leading cause of death and disability due to acute myocardial infarction, angina pectoris, or ischemic heart failure (Mendis et al., 2011). The main therapeutic intervention for IHD consists of thrombolytic therapy or primary percutaneous coronary intervention, which aims to reduce myocardial infarct size and improve clinical outcomes. However, the reperfusion of acutely ischemic myocardium can lead to further cardiomyocyte damage, known as myocardial ischemia-reperfusion (I-R) injury, involving clinical manifestations of arrhythmia, myocardial stunning, microvascular obstruction and myocardial necrosis (Hausenloy and Yellon, 2013).

Silent information regulator 1 (Sirt1) is a nicotinamide adenine dinucleotide (NAD)-dependent histone deacetylase that performs a wide variety of functions in different biologic systems. Sirt1 plays a protective role in the pathophysiology of vascular aging and age-related diseases including neurodegenerative diseases, cardiovascular disease, chronic kidney disease, osteoporosis and the metabolic syndrome (Guarente, 2011). Research has reported that activation of Sirt1 extends the lifespan and retards heart-related aging in mice fed a high-fat diet (Mitchell et al., 2014). Previous studies have demonstrated that activation of Sirt1 signaling mimics ischemic preconditioning and protects against myocardial I-R injury (Yamamoto and Sadoshima, 2011; Ding et al., 2015; Yamamoto et al., 2016; Yu et al., 2016). After myocardial I-R injury, the extent of myocardial infarction and the number of TUNEL-positive nuclei are significantly reduced in transgenic mice displaying a cardiac-specific overexpression of Sirt1 compared with non-transgenic mice (Hsu et al., 2010). In diabetic rats, upregulation of Sirt1 in the heart improved cardiac function and reduced infarct size to the same extent as that observed in non-diabetic animals following myocardial I-R injury, and this phenomenon was associated with reductions in serum creatine kinase-MB (CK-MB), lactate dehydrogenase (LDH) activities and cardiomyocyte apoptosis (Ding et al., 2015). Sirt1 also stimulates the expression of pro-survival molecules and negatively regulates the survival of pro-apoptotic molecules through the deacetylation of the forkhead box O (FoxO) family transcription factors, reducing oxidative damage and apoptosis in Sirt1 transgenic mice with myocardial I-R injury (Hsu et al., 2010; Yu et al., 2016). Thus, modulation of Sirt1 signaling is a potential therapeutic strategy in myocardial I-R injury.

Lumbrokinase, an extract of *Lumbricus rubellus*, was identified in the early 1990s (Mihara et al., 1991) as a group of bioactive proteolytic enzymes ranging in molecular weight from 25 to 32 kDa (Cho et al., 2004) that includes plasminogen activator and plasmin (Cooper and Balamurugan, 2010). The extrinsic plasminogen activator (e-PA) in lumbrokinase is similar to endogenous tissue plasminogen activator (t-PA) found in other species. Lumbrokinase can dissolve fibrin clots and convert plasminogen to plasmin by increasing endogenous t-PA activity. Moreover, lumbrokinase enzymes show thrombolytic activity only in the presence of fibrin. Thus, lumbrokinase is not associated with the excessive bleeding and heavy blood loss seen with streptokinase or urokinase, which may result in death (Delaney et al., 2007; Vernooij et al., 2009). In our previous study, pre-treatment with lumbrokinase 10 μg/kg significantly reduced ventricular arrhythmias and myocardial infarction in rats after myocardial I-R injury (Wang et al., 2016). We suggested that lumbrokinase has significant potential as a cardioprotective agent, regulating anti-inflammatory mechanisms that protect the heart against I-R injury (Wang et al., 2016). Oral lumbrokinase supplementation has been used in Japan, Korea, Canada and the United States to support and maintain healthy cardiovascular function. In addition, lumbrokinase is used clinically as a thrombolytic agent in China in the treatment of stroke and cardiovascular disease (Wang et al., 2013), the prevention of secondary ischemic stroke (Cao et al., 2013), and for the improvement of myocardial perfusion in stable angina (Kasim et al., 2009). Lumbrokinase has approved a phase III clinical trial that indicated the total effective rate was 88.21% and the significant response rate was 68.91% in treating ischemic cerebrovascular disease. However, the underlying molecular mechanism involved in the amelioration of myocardial I-R injury by post-ischemic treatment with lumbrokinase remains unclear. The aims of the present study were (1) to explore the effects of post-ischemic treatment with lumbrokinase in rats subjected to myocardial I-R injury, (2) investigate whether lumbrokinase treatment confers anti-inflammation, anti-oxidative and anti-apoptotic effects in myocardial I-R injury, and (3) examine whether Sirt1 signaling ameliorates myocardial I-R injury in rats.

## Materials and Methods

### Animals

Six-week-old male Sprague-Dawley rats (LASCO Co., Charles River Technology, Taipei, Taiwan) weighing 250–300 g were housed in the Chung Shan Medical University Animal Center at an ambient temperature of 25 ± 1°C under a normal 12 h light-12 h dark cycle. The animals were fed with normal chow and given water *ad libitum*. All surgical procedures were reviewed and approved by the Chung Shan Medical University Institutional Animal Care and Use Committee. The study complied with the protocols outlined in the *Guide for the Care and Use of Laboratory Animals* issued in 2011 by the US National Research Council Committee. The animal study protocol was approved by the Institutional Animal Ethics Committee of Chung Shan Medical University, Taichung, Taiwan (IACUC 1431). All efforts were taken to minimize animal suffering and the numbers of sacrificed animals.

### Myocardial Ischemia-Reperfusion Injury Model

Myocardial I-R injury was induced by temporary occlusion of the left anterior descending coronary artery, according to a previously described procedure (Wang et al., 2016). Slight modification of the procedure meant that the left anterior descending coronary artery was occluded by tightening of the ligature to induce ischemia for 30 min, followed by 3 h of reperfusion.

### Experimental Groups

For this study, we referred to the effective dose of lumbrokinase used in our previous publication (Wang et al., 2016). Lumbrokinase (10 μg/kg; Canada RNA Biochemical Inc. Richmond, BC, Canada) or vehicle (sterile saline) was intravenously infused 20 min after occlusion of the left anterior decending coronary artery. The selective Sirt1 inhibitor EX527 (Sigma-Aldrich, St Louis, MO, United States) was intraperitoneally injected at a dose of 5 mg/kg 15 min after coronary artery occlusion (CAO). The dose of EX527 was based on previous study (Yu et al., 2017). The animals were randomly assigned to the following groups: (1) Sham-operated group (Sham); (2) Myocardial I-R+vehicle group (Vehicle); (3) Myocardial I-R+lumbrokinase group (LK); (4) Myocardial I-R+LK + EX527 group (LK+EX527).

### Evaluation of Arrhythmias and Cardiac Function

Antiarrhythmic effects of lumbrokinase were evaluated during myocardial I-R injury, using the diagnostic criteria recommended by the Lambeth Convention (Curtis et al., 2013). The incidence and duration of ventricular tachycardia (VT) and ventricular fibrillation (VF) were determined in both surviving rats and those that died. In rats with irreversible VF, the duration of VF was recorded until mean BP was less than 15 mmHg. To evaluate the effect of lumbrokinase on cardiac function during myocardial I-R injury, a Millar catheter was inserted into the left ventricular cavity via the right common carotid artery and changes in the left ventricular systolic pressure (LVSP), left ventricular diastolic pressure (LVDP) and maximal slope of systolic pressure increment (max dP/dt) and diastolic decrement (min dP/dt) were continuously recorded using a Transonic Scisense Pressure Measurement system (SP200, Transonic Scisense Inc., Ontario, Canada).

### Determination of Myocardial Infarct Size

Myocardial infarct size was determined by the double-staining technique using Evans blue and 2,3,5-triphenyltetrazolium chloride (TTC; Sigma-Aldrich, St Louis, MO, United States) (Bohl et al., 2009). At the end of the experiment, the coronary artery was re-occluded and 1% Evans blue solution was intravenously injected to stain non-ischemic myocardium and determine the area at risk. The heart was cut transversely into 2-mm-thick slices using a heart slicer matrix (Jacobowitz Systems, Zivic-Miller Laboratories Inc., Allison Park, PA, United States). Heart sections were stained with 2% TTC at 37°C for 30 min in darkness. The slices were placed in a solution with 10% formalin at room temperature for 1 day. Infarcted tissue slices were scanned and tissue weights were evaluated by distinguishing the normal myocardium (stained blue) from the area at risk and infarct area (unstained) in a TTC staining assay.

### Determination of Myocardial Damage

Arterial blood collected from the carotid catheter in rats that survived after 30 min of ischemia and 3 h of reperfusion was centrifuged at 3,000 g for 10 min to isolate plasma and determine myocardial damage. Myocardial cellular damage was determined using automated clinical analyzers to measure plasma activities of LDH and CK-MB (ADVIA 1800, Siemens Healthcare Diagnostics Inc., NY, United States) and the levels of troponin I (Centaur, Siemens Healthcare Diagnostics Inc., NY, United States).

### Protein Extraction and Western Blot Analysis

Myocardium samples from surviving rats at the end of experiment were homogenized with a tissue protein extraction reagent (Thermo Scientific, United States) containing a protease inhibitor cocktail (Sigma-Aldrich, St Louis, MO, United States). The homogenates were then centrifuged at 12,000 g for 10 min at 4°C. The supernatant was mixed with an equal volume of loading buffer and heated at 95°C for 10 min. The protein samples were subjected to SDS-PAGE and electrophoretically transferred onto PVDF protein sequencing membranes for 90 min. The membrane was blocked with 5% non-fat milk in PBS with 0.1% (v/v) Tween-20 (PBST) at room temperature for 1 h. The membrane was washed and blotted with the antibodies of Bax, Bcl-2, Beclin-1 (Abcam, United Kingdom), succinate dehydrogenase (SDH), cytochrome c oxidase (CcO), Caspase-3, Cleaved-Caspase-3, p-NF-κB, NF-κB, iNOS, FoxO1 (Cell Signaling, United States), Sirt1, Ac-FoxO1, manganese-dependent superoxide dismutase (MnSOD) (Santa Cruz, United States), COX-2 (Cayman Chemical, United States), LC3, p62, and β-actin (Novus, United States). The membrane was incubated with HRP-conjugated secondary antibody (Jackson ImmunoResearch Laboratories, United States) prior to chemiluminescence detection (Pierce, United States). Western blot analyses of protein expression were performed using two different samples from each treatment group and repeated at least three times (using the same samples), we thus obtained at least six values for each study group. And then, chose the lower value of sham group as standard, each of band intensity divide by the standard and multiplied by 100 to get % of sham.

### Statistical Analysis

Data are expressed as the mean ± standard error of the mean (SEM). Differences between the groups in myocardial I-R-induced infarction, duration of arrthymias, cardiac function, and Western blot data were compared using one-way analysis of variance (ANOVA) and the variables were subsequently analyzed using Bonferroni’s tests to determine any significant between-group differences. Between-group differences in VT and VF percentages were analyzed using the χ2 test and a significance level was set at 0.05 for each comparison.

## Results

### Effects of Post-ischemic Treatment With Lumbrokinase on Cardiac Function

**FIGURE 1:**
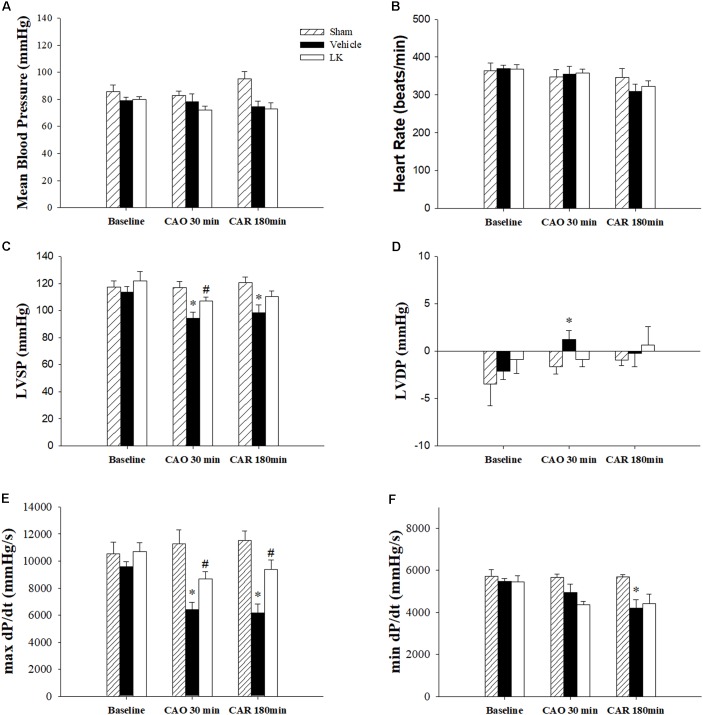
Cardiac function parameters in I-R rats treated with vehicle or lumbrokinase. Post-ischemic treatment with vehicle or lumbrokinase (10 μg/kg) in rats subjected to myocardial I-R injury. **(A)** Mean blood pressure, **(B)** heart rate, **(C)** LVSP, **(D)** LVDP, **(E)** max dP/dt, and **(F)** min dP/dt were determined before CAO (baseline), 30 min after CAO (CAO 30 min), and 180 min after reperfusion (CAR 180 min). Each value is represented as the mean ± SEM (*n* = 4–8). ^∗^*p* < 0.05 compared with the sham group; ^#^*p* < 0.05 compared with the vehicle group.

The effects of post-ischemic lumbrokinase treatment on myocardial I-R-induced cardiac dysfunction are shown in **Figure 1**. HR and MBP did not differ significantly amongst the groups. We measured LVSP, LVDP, max dP/dt and min dP/dt to detect cardiac systolic and diastolic function parameters. Baseline cardiac function parameters did not differ significantly amongst the sham-operated group, vehicle group and LK group. As expected, LVSP, LVDP, max dP/dt, and min dP/dt in the vehicle group were significantly worse after 30 min of myocardial ischemia followed by 180 min of reperfusion, compared with measurements in the sham group. Post-ischemic lumbrokinase treatment significantly reversed cardiac function injury by significantly increasing the LVSP and max dP/dt after 30 min of myocardial ischemia and significantly increasing max dP/dt after myocardial I-R injury, compared with the vehicle group. We therefore suggest that post-ischemic treatment with lumbrokinase preserves cardiac function after myocardial I-R injury.

### Effects of Post-ischemic Treatment With Lumbrokinase on Myocardial I-R-Induced Myocardial Damage and Rhythm Disturbances

The effects of post-ischemic treatment with lumbrokinase on infarct size after myocardial I-R injury were determined by Evans-blue/TTC staining. As shown in **Table 1**, there were no significant differences in the area at risk (AAR)/ventricle ratio amongst experimental groups, indicating that in each group, similar amounts of myocardium were at risk from left CAO. Compared to the sham group, 30 min of ischemia followed by 180 min of reperfusion resulted in severe myocardial injury. The infarct/AAR ratio was significantly reduced by post-ischemic treatment with lumbrokinase. We also measured LDH activity, a marker of tissue damage, CK-MB activity and troponin I levels, important indicators of the extent of myocardial cellular injury, to investigate the effects of post-ischemic lumbrokinase treatment on myocardial I-R-induced myocardial damage. LDH and CK-MB activities, as well as troponin I levels, were significantly higher in the vehicle group compared with the sham group. In comparison with vehicle-treated rats, LDH and CK-MB activities and troponin I levels in plasma were significantly decreased after post-ischemic lumbrokinase treatment. LDH and CK-MB activities and also troponin I levels were consistent with infarct size data.

**Table 1:** Effects of post-ischemic lumbrokinase treatment in rats subjected to myocardial I-R injury.

		Sham (*n* = 6)	Vehicle (*n* = 13)	LK (*n* = 13)
**Myocardial Infarction**		
Ventricle (g)		0.78 ± 0.07	0.87 ± 0.05	0.86 ± 0.02
AAR (g)		–	0.55 ± 0.01	0.57 ± 0.01
AAR/Ventricle (%)		–	63.8 ± 2.89	65.8 ± 0.69
Infarct (g)		–	0.13 ± 0.004	0.11 ± 0.003^#^
Infarct/AAR (%)		–	23.6 ± 0.96	19.6 ± 0.59^#^
**Cardiac Biomarkers**		
LDH (U/L)		1083.0 ± 229.6	4290.0 ± 940.4^∗^	3125.3 ± 577.4^#^
CK-MB (U/L)		4525.5 ± 1548.5	10053.6 ± 1285.9^∗^	7209.8 ± 984.9^#^
Troponin-I (ng/mL)		1.07 ± 0.68	189.29 ± 52.68^∗^	39.44 ± 19.00^#^
**Arrthymias**		
VT	Incidence (%)	–	86	46^#^
	Duration (sec)	–	26.4 ± 8.4	3.8 ± 1.8^#^
VF	Incidence (%)	–	36	8
	Duration (sec)	–	19.8 ± 8.6	0.9 ± 0.9^#^

*AAR, area at risk; LDH, lactate dehydrogenase; CK-MB, creatine kinase-MB; VT, ventricular tachycardia; VF, ventricular fibrillation. Values are shown as the mean ± SEM. ^∗^*p* < 0.05 compared with sham; ^#^*p* < 0.05 compared with vehicle*.

The effects of post-ischemic lumbrokinase treatment on myocardial I-R-induced arrhythmias are shown in **Table 1**. In the vehicle group, the incidence of VT was 86% (26.4 ± 8.4 s) and incidence of VF was 36% (19.8 ± 8.6 s) during the period of myocardial I-R injury. Post-ischemic lumbrokinase administration significantly decreased the incidence of VT to 46% and VT duration to 3.8 ± 1.8 s, and the incidence of VF to 8% and duration to 0.9 ± 0.9 s compared with the vehicle group. These results indicate that post-ischemic treatment with lumbrokinase decreases myocardial I-R-induced rhythm disturbances.

### Effects of Post-ischemic Treatment With Lumbrokinase on Sirt1 Protein Expression in Myocardial Tissue

**FIGURE 2:**
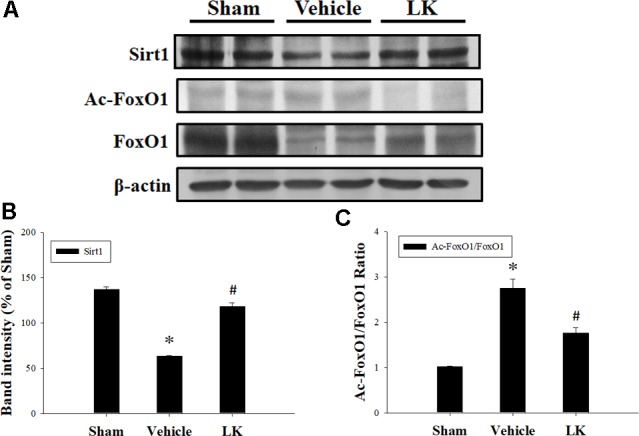
Post-ischemic lumbrokinase treatment activated Sirt1 after myocardial I-R injury. Post-ischemic treatment with vehicle or lumbrokinase (10 μg/kg) in rats subjected to myocardial I-R injury. **(A)** Representative images of the Western blot results. Quantitative densitometric analysis of **(B)** Sirt1 protein expression and **(C)** the ratio of Ac-FoxO1 to FoxO1 in rat myocardium after myocardial I-R injury. Data are normalized with values from the sham group and presented as percentage rates. Each value is represented as the mean ± SEM (*n* = 3). ^∗^*p* < 0.05 compared with the sham group; ^#^*p* < 0.05 compared with the vehicle group.

As shown in **Figure 2**, Sirt1 protein expression was significantly decreased in the vehicle group compared with the sham group. Notably, post-ischemic treatment with lumbrokinase significantly increased Sirt1 protein expression in comparison with the vehicle group. As presented in **Figure 2C**, the Ac-FoxO1/FoxO1 ratio was significantly increased in the vehicle group compared with the sham group, while post-ischemic lumbrokinase treatment significantly decreased the Ac-FoxO/FoxO1 ratio compared with the vehicle group. These results suggest that post-ischemic treatment with lumbrokinase activates Sirt1 and induces deacetylation of FoxO1.

### Effects of Post-ischemic Treatment With Lumbrokinase on Myocardial I-R-Induced Mitochondrial Oxidative Damage

**FIGURE 3:**
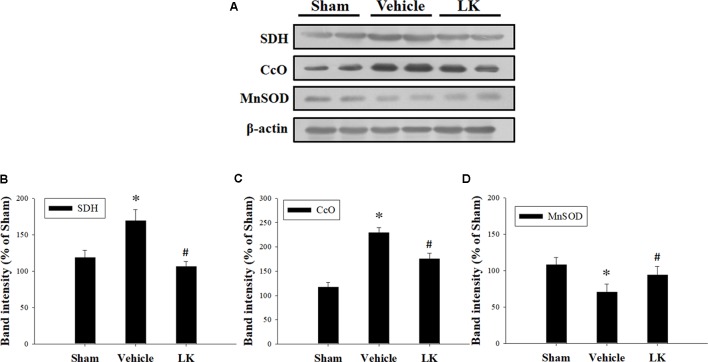
Post-ischemic lumbrokinase treatment reduced mitochondrial oxidative stress induced by myocardial I-R injury. **(A)** Representative images of the Western blot results. Quantitative densitometric analysis of **(B)** SDH, **(C)** CcO, and **(D)** MnSOD protein expression in rat myocardium after myocardial I-R injury. Data are normalized with values from the sham group and presented as percentage rates. Each value is represented as the mean ± SEM (*n* = 3). ^∗^*p* < 0.05 compared with the sham group; ^#^*p* < 0.05 compared with the vehicle group.

Mitochondrial electron transport chain (ETC) deficiency induces reactive oxygen species (ROS) overproduction that may increase SDH and CcO activity in myocardial I-R injury (Muntean et al., 2016). We evaluated the role of mitochondrial oxidative damage in the cardioprotective effect of post-ischemic lumbrokinase treatment by evaluating the levels of SDH and CcO protein expression in rats subjected to myocardial I-R injury (**Figure 3**). We found that SDH and CcO levels were significantly increased after myocardial I-R injury compared with those in the sham group. Post-ischemic lumbrokinase treatment significantly attenuated SDH and CcO expression. We also assessed MnSOD protein expression to examine the effects of post-ischemic lumbrokinase treatment on oxidative stress (**Figure 3**). We found that MnSOD expression was significantly decreased after myocardial I-R injury compared with the sham-operated group. Post-ischemic lumbrokinase treatment significantly increased MnSOD expression compared with levels in the vehicle group. These results indicate that post-ischemic treatment with lumbrokinase attenuates myocardial I-R-induced mitochondrial oxidative damage.

### Effects of Post-ischemic Treatment With Lumbrokinase on Myocardial I-R-Induced Cardiomyocyte Apoptosis

**FIGURE 4:**
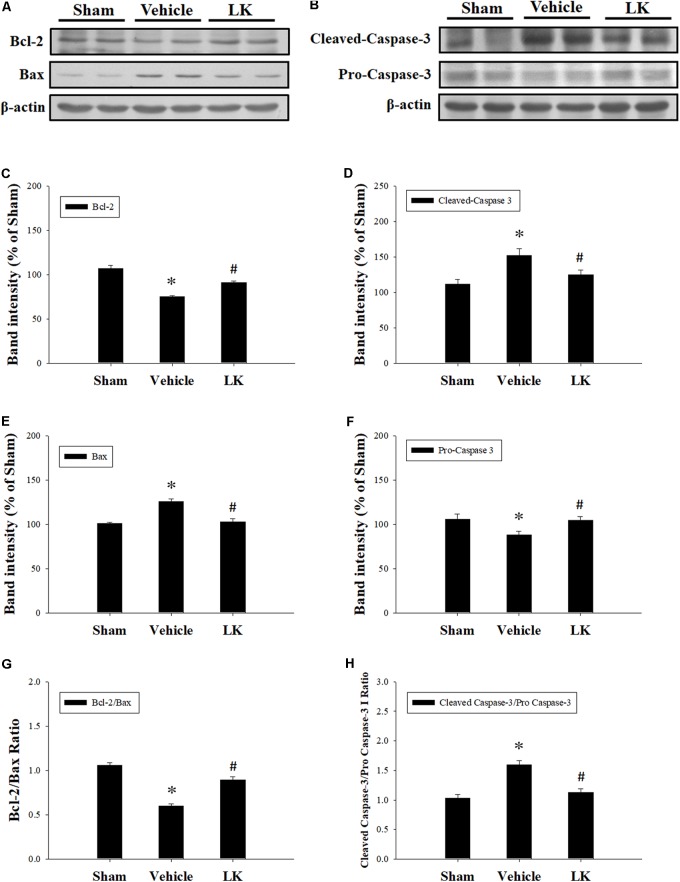
Post-ischemic lumbrokinase treatment attenuated cardiomyocyte apoptosis in rats subjected to myocardial I-R injury. Representative immunoblot images of **(A)** Bcl-2 and Bax, and **(B)** Cleaved-Caspase-3 and Pro-Caspase-3. Quantitative densitometric analysis showing expression of **(C)** Bcl-2, **(D)** Bax, **(E)** the ratio of Bcl-2 to Bax, **(F)** Cleaved-Caspase 3 **(G)** Pro-Caspase-3 and **(H)** the ratio of Cleaved-Caspase 3 to Pro-Caspase-3 in rat myocardium after myocardial I-R injury. Data are normalized with values from the sham group and presented as percentage rates. Each value is represented as the mean ± SEM (*n* = 3). ^∗^*p* < 0.05 compared with the sham group; ^#^*p* < 0.05 compared with the vehicle group.

In order to investigate the anti-apoptotic effects of post-ischemic lumbrokinase treatment, we evaluated the expression of Bcl-2, Bax and Caspase-3 in rats subjected to myocardial I-R injury (**Figure 4**). Compared with the sham-operated group, the expression of Bax, the active form of caspase-3 and the Cleaved-Caspase-3/Pro-Caspase-3 ratio were significantly increased, while Bcl-2 expression and the Bcl-2/Bax ratio were significantly decreased in the vehicle group. The opposite trends were observed with post-ischemic lumbrokinase administration, with significant reductions in Bax, Cleaved-Caspase-3 expression and the Cleaved-Caspase-3/Pro-Caspase-3 ratio, and significant increases in Bcl-2 expression and the Bcl-2/Bax ratio in the myocardium. These results indicate that post-ischemic treatment with lumbrokinase attenuates myocardial I-R-induced cardiomyocyte apoptosis.

### Effects of Post-ischemic Treatment With Lumbrokinase on Myocardial I-R-Induced Inflammation

**FIGURE 5:**
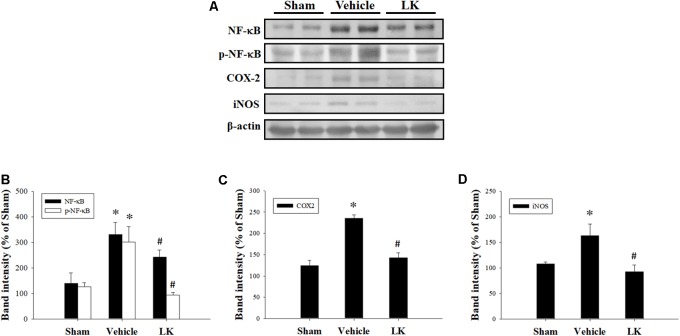
Post-ischemic lumbrokinase treatment reduced levels of inflammation induced by myocardial I-R injury. **(A)** Representative images of the Western blot results. Quantitative densitometric analysis of **(B)** NF-κB and p-NF-κB, **(C)** COX-2, and **(D)** iNOS protein expression in rat myocardium after myocardial I-R injury. Data are normalized with values from the sham group and presented as percentage rates. Each value is represented as the mean ± SEM (*n* = 3). ^∗^*p* < 0.05 compared with the sham group; ^#^*p* < 0.05 compared with the vehicle group.

We measured the protein expression levels of NF-κB, p-NF-κB, COX-2, and iNOS to assess the role played by inflammation on the effect of lumbrokinase post-ischemic treatment in rats subjected to myocardial I-R injury. As shown in **Figure 5**, we found significant increases in NF-κB, p-NF-κB, COX-2, and iNOS protein expression in the myocardium after myocardial I-R injury compared with the sham-operated animals. Levels of NF-κB, p-NF-κB, COX-2, and iNOS expression were significantly decreased by post-ischemic lumbrokinase treatment compared with levels in vehicle-treated animals. These results indicate that post-ischemic treatment with lumbrokinase attenuates myocardial I-R-induced inflammation.

### Effects of Post-ischemic Treatment With Lumbrokinase on Myocardial I-R-Induced Autophagy

**FIGURE 6:**
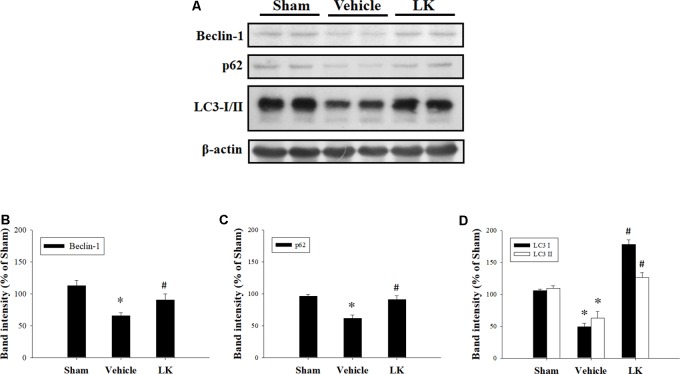
Post-ischemic lumbrokinase treatment enhanced autophagic flux in rats subjected to myocardial I-R injury. **(A)** Representative images of the Western blot results. Quantitative densitometric analysis of **(B)** Beclin-1, **(C)** p62, and **(D)** LC3-I/II protein expression in rat myocardium after myocardial I-R injury. Data are normalized with values from the sham group and presented as percentage rates. Each value is represented as the mean ± SEM (*n* = 3). ^∗^*p* < 0.05 compared with the sham group; ^#^*p* < 0.05 compared with the vehicle group.

We evaluated the levels of Beclin-1, p62, and LC3 expression to examine the role of autophagy in the effect of post-ischemic lumbrokinase treatment (**Figure 6**). We found that Beclin-1, p62, and LC3 expression were all significantly decreased after myocardial I-R injury compared with the sham-operated group, and that post-ischemic lumbrokinase treatment significantly increased the levels of these autophagy-related proteins after myocardial I-R injury compared with those in the vehicle group. These results indicate that post-ischemic lumbrokinase treatment enhances autophagy.

### Effects of Post-ischemic Treatment With Lumbrokinase and EX527 in Rats Subjected to Myocardial I-R Injury

**FIGURE 7:**
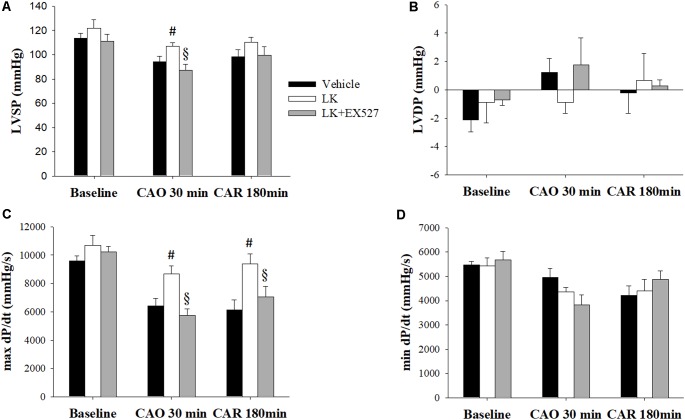
Post-ischemic lumbrokinase treatment reserved myocardial ischemia-reperfusion injury induced cardiac dysfunction via Sirt1 activation. Post-ischemic treatment with vehicle or lumbrokinase (10 μg/kg) was given 20 min after coronal artery ligation; the Sirt1 specific inhibitor, EX527, was administered 15 min after coronal artery ligation. Cardiac function parameters were assessed by a pressure transducer catheter inserted into the LV via the carotid artery. **(A)** LVSP, **(B)** LVDP, **(C)** max dP/dt, and **(D)** min dP/dt were determined before CAO (baseline), 30 min after CAO (CAO 30 min), and 180 min after reperfusion (CAR 180 min). Each value is represented as the mean ± SEM (*n* = 4–10). ^#^*p* < 0.05 compared with the vehicle group; ^§^ *p* < 0.05 compared with the LK group.

**FIGURE 8:**
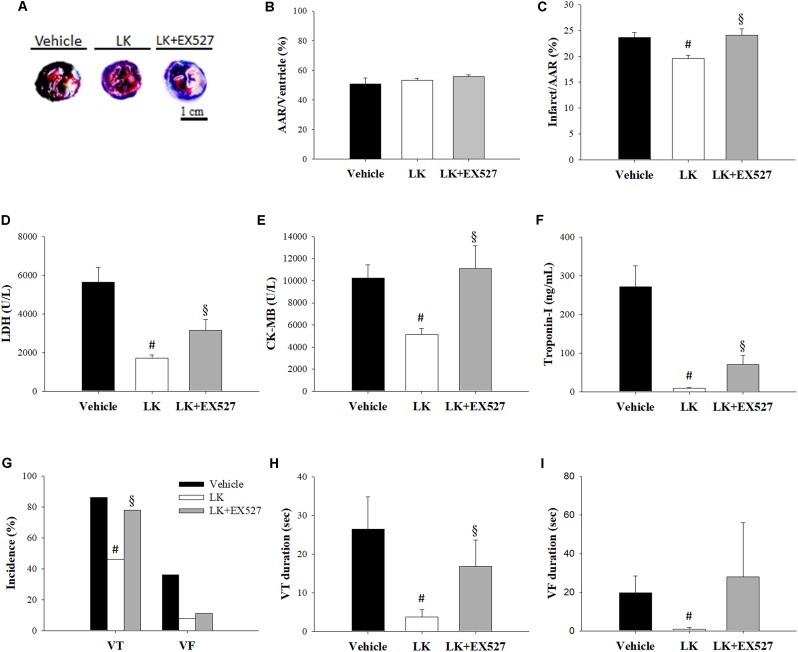
Post-ischemic lumbrokinase treatment attenuated myocardial damage via the Sirt1 signaling pathway. Myocardial infarct size was evaluated by Evans blue/TTC staining. **(A)** Representative images of heart horizontal sections. The blue area depicts non-ischemic tissue, red plus white depicts at-risk tissue, white depicts infarcted tissue. **(B)** The area at risk (AAR) was measured relative to the ventricle and **(C)** the infarct was measured relative to the AAR. The activities of **(D)** LDH, **(E)** CK-MB, and the levels of **(F)** troponin-I were assessed by an automated clinical analyzer. **(G)** The incidence of VT and VF and the duration of **(H)** VT and **(I)** VF were monitored during 30 min of ischemia followed by 3 h of reperfusion. Each value is represented as the mean ± SEM (*n* = 4–13). ^#^*p* < 0.05 compared with the vehicle group; ^§^ *p* < 0.05 compared with the LK group.

Further to the above-mentioned results, we speculated that the post-ischemic cardioprotective effects of lumbrokinase are associated with Sirt1 signaling. We therefore examined the effects of EX527, a selective Sirt1 inhibitor, to investigate the mechanisms underlying lumbrokinase-induced effects. Firstly, the effects of post-ischemic treatment with lumbrokinase and EX527 on myocardial I-R-induced cardiac dysfunction are shown in **Figure 7**. Baseline cardiac function parameters did not differ significantly amongst these groups. In the LK, LVSP and max dP/dt values at CAO 30 min and the value of max dP/dt at 180 min after coronary artery reperfusion (CAR 180 min) were significantly increased compared with the vehicle group. In rats intraperitoneally injected with EX527 at 5 min before lumbrokinase administration, LVSP and max dP/dt values at CAO 30 min and max dP/dt at CAR 180 min were significantly decreased compared with those in the LK. Secondly, the influences of post-ischemic treatment with lumbrokinase and EX527 on myocardial I-R-induced myocardial damage are presented in **Figure 8**. In comparison with vehicle-treated rats, post-ischemic lumbrokinase treatment significantly reduced the infarct/AAR ratio, LDH, and CK-MB activities and troponin I levels in plasma collected at the end of the myocardial I-R procedure. In addition, we found that EX527 treatment abolished the protective effects of lumbrokinase by significantly increasing the infarct/AAR ratio, LDH, and CK-MB activities and the troponin levels in plasma determined after myocardial I-R, compared with the LK. Thirdly, the outcomes of lumbrokinase and EX527 on myocardial I-R-induced arrhythmias in rats are expressed in **Figure 8**. Post-ischemic administration with lumbrokinase significantly reduced the incidence of VT and the durations of VT and VF compared with the vehicle group. The anti-arrythmic effect of lumbrokinase was abolished by administration of EX527, which significantly increased the incidence of VT to 78% and the duration of VT to 16.8 ± 6.8 s compared with values in theLK. Based on the above results, we suggest that the cardioprotective effects of post-ischemic treatment with lumbrokinase may involve the Sirt1-related signaling pathway.

## Discussion and Conclusion

**FIGURE 9:**
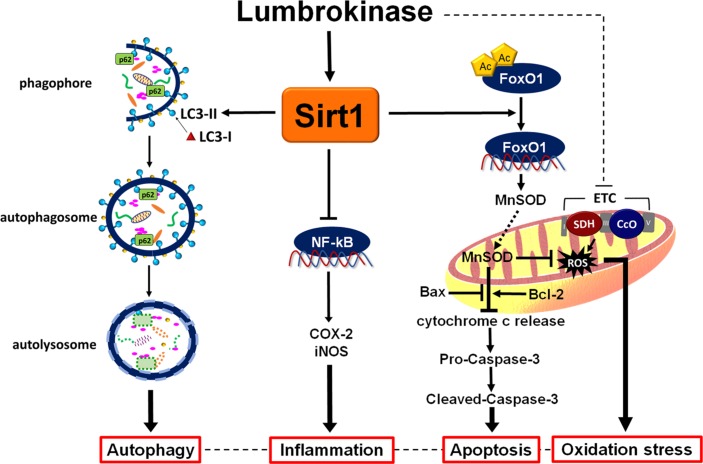
Schematic diagram of the effects of post-ischemic lumbrokinase treatment in myocardial I-R injury. Post-ischemic lumbrokinase treatment attenuates myocardial I-R injury through the activation of Sirt1 signaling, inhibition of NF-κB expression and activation of FoxO1 and LC3, and thus reduces inflammation, oxidative stress and apoptosis, and enhances autophagy after myocardial I-R injury.

The important findings of this study are as follows: first, we found that administration of lumbrokinase 20 min after ischemia significantly reduced the durations of VT and VF and the incidence of VT, improved cardiac function and decreased myocardial infarction, all of which are consistent with our findings that post-ischemic lumbrokinase treatment decreases plasma LDH, CK-MB activities and troponin I levels, which serve as indicators of myocardial damage. Second, we have demonstrated that post-ischemic lumbrokinase treatment decreased oxidative stress, inflammation and apoptosis after myocardial I-R injury. Lumbrokinase also increased autophagosome levels and activated Sirt1, which induced the deacetylation of FoxO1. Third, our results indicate that EX527, a specific Sirt1 inhibitor, abolishes the cardioprotective effects of lumbrokinase. Importantly, this is the first study to suggest that Sirt1 signaling plays a critical role in the cardioprotective effects of lumbrokinase when administered after myocardial ischemia. The data in **Figure 9** summarize the effect of post-ischemic treatment with lumbrokinase in rats subjected to myocardial I-R injury.

Previous studies have demonstrated that the reperfusion of ischemic myocardium induces the excessive generation of ROS, resulting in oxidative stress (Zhou et al., 2015). Mitochondrial ETC have been conventionally recognized as the major source of ROS, activating cytotoxic mechanisms responsible for cell death via apoptosis and necrosis during myocardial I-R injury (Brown and Griendling, 2015). ETC components are organized into five complexes (complex I, II, III, IV, and V), with each complex containing several different electron carriers. Although the main sources of ROS in the myocardial mitochondria are complexes I and III (Chen and Zweier, 2014), some studies have indicated that inhibition of SDH (complex II) or CcO (complex IV) may also reduce ROS generation (Drose et al., 2011; Chouchani et al., 2014; Yang et al., 2017). Our results show that levels of SDH and CcO expression are significantly increased in the myocardium after myocardial I-R injury. Post-ischemic lumbrokinase treatment significantly attenuated these increases. Interestingly, we also observed in this myocardial I-R injury model that MnSOD expression was significantly decreased in the myocardium after myocardial I-R injury and that post-ischemic lumbrokinase treatment significantly reversed this phenomenon. MnSOD is the main endogenous antioxidant enzyme that protects the heart from active oxygen species (Csanyi and Miller, 2014). This protection is necessary for preservation of ETC function and certain functions of the tricarboxylic acid (TCA) cycle. MnSOD is not only an essential cytoprotector against the deleterious effects of ROS but is also a mediator of mitochondrial ROS signaling through the generation of H_2_O_2_. Changes in MnSOD activity are associated with a variety of diseases, such as cardiovascular disease, cancer, the metabolic syndrome and the process of aging (Candas and Li, 2014).

It is well known that an imbalance between ROS production and elimination leads to inflammatory reaction in myocardial I-R injury (Xiong et al., 2010; Rodrigo et al., 2013). Subsequent ROS generation by polymorphonuclear neutrophils (PMNs) triggers inflammation, causing endothelial dysfunction and severe cardiac damage (Mittal et al., 2014; Valle Raleigh et al., 2017). The activation of the NF-κB signaling pathway plays a central role in the inflammatory processes that occur during myocardial I-R injury (Kitada et al., 2016). When exposed to I-R injury, various cytokines are released that trigger an inflammatory reaction and ROS overproduction leading to oxidative stress, which activates cytotoxic mechanisms responsible for cardiac dysfunction and cell death via apoptosis and necrosis (Yellon and Hausenloy, 2007; Steffens et al., 2009; Padfield et al., 2013; Yang et al., 2013; Brown and Griendling, 2015). In this study, we found that post-ischemic lumbrokinase treatment induces cardioprotective effects that are partly mediated by reducing the inflammatory response. The results of this present study show that post-ischemic lumbrokinase treatment in myocardial I-R injury significantly reduces levels of NF-κB, COX-2, and iNOS expression in the I-R myocardium. These findings suggest that post-ischemic treatment with lumbrokinase reduces inflammation after myocardial I-R injury.

Apoptosis is a process of programmed cell death that occurs in multicellular organisms, which is initiated shortly after the onset of myocardial infarction and becomes significantly enhanced during reperfusion (Gu et al., 2014). The Bcl-2 family of proteins acts as a critical checkpoint of apoptotic cell death. The anti-apoptotic members of this family, such as Bcl-2, interact with the pro-apoptotic members, such as Bax, which is required to regulate the process of cytochrome *c* release from the mitochondrion and modulate sensitivity to cell death signals (Letai, 2005). Caspases are a family of protease enzymes that are crucial mediators of programmed cell death. Initiator caspases activate executioner caspases that subsequently coordinate their activities to cleave the nuclear lamin and activate other enzymes (McIlwain et al., 2013). Caspase-3 is considered to be the most important of the executioner caspases. Once caspases are initially activated, there seems to be an irreversible commitment towards cell death (Elmore, 2007). In this study, we found that post-ischemic lumbrokinase treatment provided anti-apoptotic effects by increasing the Bcl-2/Bax ratio and decreasing the Cleaved-Caspase-3/Pro-Caspase-3 ratio after myocardial I-R injury. Notably, apoptosis and autophagy are important in cellular programmed death that occurs in myocardial I-R injury. Apoptosis can regulate autophagy; conversely, autophagy can regulate apoptosis (Gordy and He, 2012; Yonekawa and Thorburn, 2013). Autophagy is an important switch that protects cells from undergoing apoptosis (Green and Levine, 2014). LC3, p62, and Beclin-1 are the main autophagic indicators and are essential components for the formation of autophagosomes. At an early stage of autophagy, Beclin-1 is involved in the nucleation phase of phagopore formation, while the cytosolic LC3-I form is converted into the lipidated LC3-II form associated with autophagosomal membranes, and p62 together with ubiquitinated proteins are transported to autophagosomes (Mizushima et al., 2010). Monitoring the conversion of LC3 is considered to be one of the most reliable ways to monitor autophagic flux, preferable to analysis of other components of the autophagic mechanism such as members of the initiation complex (i.e., Beclin-1) or autolysosome substrates (i.e., p62) (Ma et al., 2015). Deficiency of Beclin-1 and p62 lead to increase ROS levels and enhancement of cell death, while treatment with rapamycin or overexpression of Beclin-1 induce autophagy and have been shown to play a protective role in response to I-R (Gurusamy et al., 2009). In this study, we found that post-ischemic lumbrokinase treatment significantly increased autophagosome levels by increasing the levels of Beclin-1, p62, and LC3 expression after myocardial I-R injury. These results illustrate that post-ischemic lumbrokinase treatment enhances autophagy and thereby attenuates myocardial I-R-induced cardiomyocyte apoptosis.

Our results also show that post-ischemic administration with lumbrokinase increased Sirt1 expression and decreased the Ac-FoxO1/FoxO1 ratio. Sirt1 has the ability to deacetylate FoxO, leading to an upregulation in expression of genes involved in cell-protective processes. Researchers have reported that Sirt1 activation reduces oxidative stress and maintains mitochondrial function by deacetylating and activating some of the FoxO family proteins and synthesizing antioxidant enzymes, such as MnSOD and catalase (Brunet et al., 2004; El Assar et al., 2013). Furthermore, Sirt1 plays a beneficial role in myocardial I-R injury (Hsu et al., 2010; Yang et al., 2013). Not only does Sirt1 activation protect against myocardial I-R injury by upregulating antioxidants and anti-apoptotic molecules, but Sirt1 also downregulates pro-apoptotic molecules through the deacetylation of FoxO1 in Sirt1 transgenic mice (Hsu et al., 2010). In this study, treatment with lumbrokinase after ischemia significantly increased Sirt1 expression. Importantly, the upregulation of Sirt1 induced by post-ischemic lumbrokinase treatment reduced levels of the pro-apoptotic factor Bax and the apoptosis-related Cleaved-Caspase-3 protein, and increased anti-apoptotic Bcl-2 expression. Notably, inhibiting Sirt1 with EX527 abolished the cardioprotective effects of lumbrokinase. We therefore suggest that Sirt1 signaling is involved in the post-ischemic cardioprotective effects of lumbrokinase treatment.

Many mechanisms of lumbrokinase have been reported in the literatures. Lumbrokinase inhibits second-hand smoke-induced apoptotic signaling and cardiac fibrosis by blocking transforming growth factor beta (TGF-β) receptors and suppressing ERK1/2 activation, and thus modulates downstream molecular events (Liao et al., 2015). Lumbrokinase also reduces side-stream cigarette smoke-induced hippocampus apoptosis and autophagy by enhancing eNOS expression and inhibiting proinflammatory NF-κB/iNOS/COX-2 signaling activity (Huang et al., 2013). Previous studies have demonstrated protective effects of lumbrokinase against I-R injury. For instance, lumbrokinase has a neuroprotective effect via its anti-platelet activity, increasing c-AMP levels and reducing calcium release from calcium stores, and acts as an anti-thrombotic by inhibiting ICAM-1 expression, and displays anti-apoptotic activity via the activation of the JAK1/STAT1 pathway in the focal cerebral ischemia injury model (Ji et al., 2008). In our previous study, the cardioprotective effects of pretreatment with lumbrokinase appeared to correlate with its anti-inflammatory effects on I-R-induced expression of COX-2, iNOS, and MMP-9, which were mediated by TLR4 signaling through the JNK and NF-κB pathways (Wang et al., 2016). According to the results in this study, we consider that Sirt1 signaling plays a critical role in the cardioprotective effects of lumbrokinase when administered after myocardial ischemia. We observed that the post-ischemic lumbrokinase treatment attenuated myocardial I-R injury by activating Sirt1 signaling, and thus reduced I-R-induced levels of COX-2, iNOS, and NF-κB expression. Evidence suggests that Sirt1 regulates inflammatory responses through NF-κB deacetylation, and that TLR4 signaling pathways culminated in activation of the transcription factor NF-κB, which controls some factors involved in inflammation, such as proinflammatory cytokines (TNF-α and IL-1β), adhesion molecules, and enzymes such as iNOS and COX-2 (Yang et al., 2012; Fuentes-Antras et al., 2014). We speculate that NF-κB plays an important regulatory role in the crosstalk between anti-inflammatory action seen with lumbrokinase when given before or after myocardial I-R injury.

In conclusion, this study demonstrates that Sirt1 is a critical regulator in the post-ischemic cardioprotective effects of lumbrokinase in rats subjected to myocardial I-R injury. We provide evidence that post-ischemic lumbrokinase administration significantly attenuates infarct size, and improves ventricular arrhythmias and cardiac dysfunction resulting from myocardial I-R injury. Moreover, post-ischemic treatment with lumbrokinase activates Sirt1 and induces FoxO1 deacetylation, which enhances autophagic flux and reduces oxidative damage, inflammation and apoptosis after myocardial I-R-injury. In our previous study, we reported that pretreatment with lumbrokinase protects the heart through anti-inflammatory mechanisms against myocardial I-R injury (Wang et al., 2016). We believe that the use of lumbrokinase will provide a novel therapeutic strategy against myocardial I-R injury. It would be very interesting to explore the therapeutic effects of this dietary supplement in the amelioration of myocardial I-R injury in humans.

## Author Contributions

Y-HW and S-SH designed research. S-AL, C-HH, H-HS, and Y-HC conducted research and analyzed data. Y-HW, JC, and S-SH wrote the paper. All authors read and approved the final manuscript.

## Conflict of Interest Statement

The authors declare that the research was conducted in the absence of any commercial or financial relationships that could be construed as a potential conflict of interest.
